# Mortality and its determinants among patients attending in emergency departments

**DOI:** 10.1186/s12873-024-01050-6

**Published:** 2024-07-19

**Authors:** Mengistu Abebe Messelu, Baye Tsegaye Amlak, Gebrehiwot Berie Mekonnen, Asnake Gashaw Belayneh, Sosina Tamre, Ousman Adal, Tiruye Azene Demile, Yeshimebet Tamir Tsehay, Alamirew Enyew Belay, Henok Biresaw Netsere, Wubet Tazeb Wondie, Gebremeskel Kibret Abebe, Sileshi Mulatu, Temesgen Ayenew

**Affiliations:** 1 Department of Nursing, College of Medicine and Health Sciences, Debre Markos University, Debre Markos, Ethiopia; 2 Department of Pediatrics and Child Health Nursing, College of Medicine and Health Sciences, Debre Tabor University, Debre Tabor, Ethiopia; 3 Department of Emergency and Critical Care Nursing, College of Medicine and Health Sciences, Bahir Dar University, Bahir Dar, Ethiopia; 4 Department of Surgical Nursing, School of Nursing, College of Medicine and Health Sciences, University of Gondar, Gondar, Ethiopia; 5 Department of Surgical Nursing, College of Medicine and Health Sciences, Bahir Dar University, Bahir Dar, Ethiopia; 6 Department of Adult Health Nursing, College of Medicine and Health Sciences, Bahir Dar University, Bahir Dar, Ethiopia; 7 Department of Pediatrics and Child Health Nursing, College of Medicine and Health Sciences, Ambo University, Ambo, Ethiopia; 8 Department of Emergency and Critical Care Nursing, College of Medicine and Health Sciences, Woldia University, Woldia, Ethiopia; 9 Department of Pediatrics and Child Health Nursing, College of Medicine and Health Sciences, Bahir Dar University, Bahir Dar, Ethiopia

**Keywords:** Associated factors, Emergency department, Ethiopia, Meta-analysis, Mortality

## Abstract

**Background:**

Due to the high burden of mortality from acute communicable and non-communicable diseases, emergency department’s mortality has become one of the major health indices in Ethiopia that should be evaluated regularly in every health institution. However, there are inconsistencies between studies, and there is no systematic review or meta-analysis study about the prevalence of mortality in the emergency department. Therefore, this study aimed to determine the pooled prevalence of mortality and identify its determinants in the emergency departments of Ethiopian hospitals.

**Methods:**

This systematic review was conducted according to the guidelines of Preferred Reporting Items for Systematic Reviews and Meta-Analyses (PRISMA) and has been registered with PROSPERO. A structured search of databases (Medline/PubMed, Google Scholar, CINAHL, EMBASE, HINARI, and Web of Science) was undertaken. All observational studies reporting the prevalence of mortality of patients in emergency departments of Ethiopian hospitals, and published in English up to December 16, 2023, were considered for this review. Two reviewers independently assess the quality of the studies using the Joanna Briggs Institute (JBI) critical appraisal tool. A meta-analysis using a random-effects model was performed to estimate the pooled prevalence. The heterogeneity of studies was assessed using I^2^ statistics, and to identify the possible causes of heterogeneity, subgroup analysis and meta-regression were used. Egger’s test and funnel plots were used to assess publication bias. STATA version 17.0 software was used for all the statistical analyses. A p-value less than 0.05 was used to declare statistical significance.

**Results:**

A total of 1363 articles were retrieved through electronic search databases. Subsequently, eighteen studies comprised 21,582 study participants were included for analysis. The pooled prevalence of mortality among patients in the Emergency Department (ED) was 7.71% (95% CI: 3.62, 11.80). Regional subgroup analysis showed that the pooled prevalence of mortality was 16.7%, 12.89%, 10.28%, and 4.35% in Dire Dawa, Amhara, Oromia, and Addis Ababa, respectively. Moreover, subgroup analysis based on patients’ age revealed that the pooled prevalence of mortality among adults and children was 8.23% (95% CI: 3.51, 12.94) and 4.48% (95% CI: 2.88, 6.08), respectively. Being a rural resident (OR; 2.30, 95% CI: 1.48, 3.58), unconsciousness (OR; 3.86, 95% CI: 1.35, 11.04), comorbidity (OR; 2.82, 95% CI: 1.56, 5.09), and time to reach a nearby health facility (OR; 4.73, 95% CI: 2.19, 10.21) were determinants of mortality for patients in the emergency departments.

**Conclusion and recommendations:**

This study found that the overall prevalence of mortality among patients in emergency departments of Ethiopian hospitals was high, which requires collaboration between all stakeholders to improve outcomes. Being a rural resident, unconsciousness, comorbidity, and time elapsed to reach health facilities were determinants of mortality. Improving pre-hospital care, training healthcare providers, early referral, and improving first-line management at referral hospitals will help to reduce the high mortality in our country.

## Background

The emergency department provides services for patients with conditions that are life-threatening or potentially life-threatening over 24 h a day and 365 days a year [1]. It is considered the backbone of health facilities and the general public by providing the first line of care upon arrival [2]. Patient mortality in Emergency Departments (ED) is a major public health problem that has aggravated in recent years [3].

The global estimate of ED mortality was 15–16%, and most of the ED deaths occur in low- and middle-income countries with scarce resources [4, 5]. The major causes of this mortality are traffic accidents, cardiovascular disease, trauma, and respiratory problems [3, 6–8]. Most emergency department mortality occurred within the first three days; the majority of these deaths are avoidable with proper intervention [8].

The World Health Organization (WHO) recommends all countries, regardless of resources or economic development, to start by establishing a comprehensive emergency care system and to monitor progress on a regular basis [9]. Because of this, emergency services are receiving more attention in Africa, where the burden of mortality from acute communicable and non-communicable diseases is still significant [10].

According to the Ethiopian Hospital Services Transformation Guideline, emergency patients should be able to access the triage area without the hindrance of their financial capacity and/or security guard [1]. Furthermore, the Ethiopian Federal Ministry of Health (EMOH) has striven to improve access to emergency care through sustainable, effective, and quality care provided by trained healthcare providers [1, 11]. Emergency room mortality has to be one of the emergency medical services indicators in Ethiopia, which should be lower than 1.5% by 2024 [1]. However, there are inconsistencies between studies, and there is no systematic review or meta-analysis study that reveals the pooled prevalence of mortality among emergency department patients. Therefore, this review and meta-analysis aimed to synthesize relevant and reliable evidence on the prevalence and determinant factors of mortality in emergency departments in Ethiopia.

## Methods

### Protocol and registration

The plan for conducting this review has been pre-registered with the International Prospective Register of Systematic Reviews (PROSPERO) under the unique identification number (UIN): review registry CRD42023493897 (https://www.crd.york.ac.uk/prospero/#myprospero). This systematic review and meta-analysis has been reported in line with the preferred Reporting Item for Systematic Review and Meta-analysis (PRISMA) 2020 guideline [12].

### Eligibility criteria

Observational studies (prospective and retrospective cohort, and cross-sectional) that reported the outcome (prevalence of mortality) in all emergency departments (adults, pediatrics, and obstetrics), conducted in Ethiopia, and published in English up to December 16, 2023, or grey literature sources were included in this review. Case control studies were included only for identifying the determinants of mortality, but they were omitted while determining the prevalence of mortality because, by their nature, they do not report the prevalence. Moreover, articles that didn’t report the outcome variable and studies with small sample size were excluded from this review. Once attempts were made to contact the corresponding authors of the studies to obtain the full texts, articles that did not provide full access and didn’t report the outcome (prevalence of mortality of patients in emergency departments) were excluded.

### Searching strategy

Following the PRISMA guideline, studies were searched using PubMed, Google Scholar, CINAHL, EMBASE, HINARI, and Web of Science without publication year restriction up to December 16, 2023. All published and grey literature was retrieved, critically evaluated, and assessed for inclusion in this study. Search terms used to access studies using PECO (Population (patients), Exposure (attending in emergency departments), Context (Ethiopian hospitals), Outcome (mortality)) strategy are the following: “mortality”, “mortalities”, “crude death rate”, “crude mortality rate”, “death”, “Ethiopia”, Federal Democratic Republic of Ethiopia”, “emergency department”, ‘emergency departments”, emergency “department”, “emergency ward”, “emergency hospital”, “emergency class”, “emergency units”, and “emergency unit”. These search strategies were developed using “AND” and/or “OR” Boolean operators.

### Outcomes

This review and meta-analysis primarily focus on determining the pooled prevalence of mortality in emergency departments in Ethiopia, expressed as a percentage with frequency. The calculation entails dividing the total number of patients who died by the overall number of patients visited in emergency departments, multiplied by 100. The authors utilized the Odds Ratio (OR) as an outcome measure to pinpoint determinants of mortality in emergency departments. Factors reported in at least two studies were considered for pooled analysis to increase the validity of this study.

### Data extraction

The articles identified in the literature search were first screened by title and abstracts for inclusion in the systematic review by independent reviewers (M.A.M, T.A, and B.T.A). Those studies that passed the title and abstract screening processes were eligible for full-text review. The independent reviewers (M.A.M, T.A, B.T.A, A.G, and G.B) evaluated the full text of the eligible studies for inclusion in the final analysis. The disagreement was handled based on the inclusion and exclusion criteria, and the third reviewer (T.A.D) was involved in the final decision.

Microsoft Excel 2019 was used to extract data encompassing the author name, publication year, region of the study, prevalence of mortality, sample size, study design, and population group. Two independent reviewers (M.A.M and T.A) extracted and cross-checked the extracted data for potential variations, and the discrepancies were handled by re-evaluating the full text. A third review (B.T.A) examined the extracted data to ensure its accuracy and identify any errors.

### Risk of bias assessment

The Joanna Briggs Institute (JBI) quality assessment tool [13] for prevalence studies was used to assess the quality of the included studies. The criteria were: (1) sample representativeness; (2) sample size adequacy; (3) using valid measurements; (4) using appropriate statistical analysis methods; and (5) response rates. Two reviewers (M.A.M. and T.A) assessed the quality of the included studies. The procedure was repeated whenever a disagreement occurred. Studies that scored greater than or equal to 50% of the assessment criteria were considered to have a low risk of bias.

### Data analysis

Endnote X-8 reference manager software was used to manage the selection process. STATA version 17 was used for data analysis. Potential publication bias was checked by the funnel plot [14] and Egger’s test [15]. The heterogeneity of articles was assessed using I^2^ statistics, and scores of 75%, 50%, and 20% correspond to high, moderate, and low levels of heterogeneity, respectively [16]. DerSimonian and Laird’s random effects model was used to estimate the pooled prevalence with a 95% CI of mortality of patients in emergency departments. Subgroup analysis was done using region, type of population, and age group. A meta-regression analysis using the sample size and publication year was conducted to identify the possible source of heterogeneity. The sensitivity test was done to check the effect of individual studies on the pooled estimate.

## Results

### Article selection

**Fig. 1:**
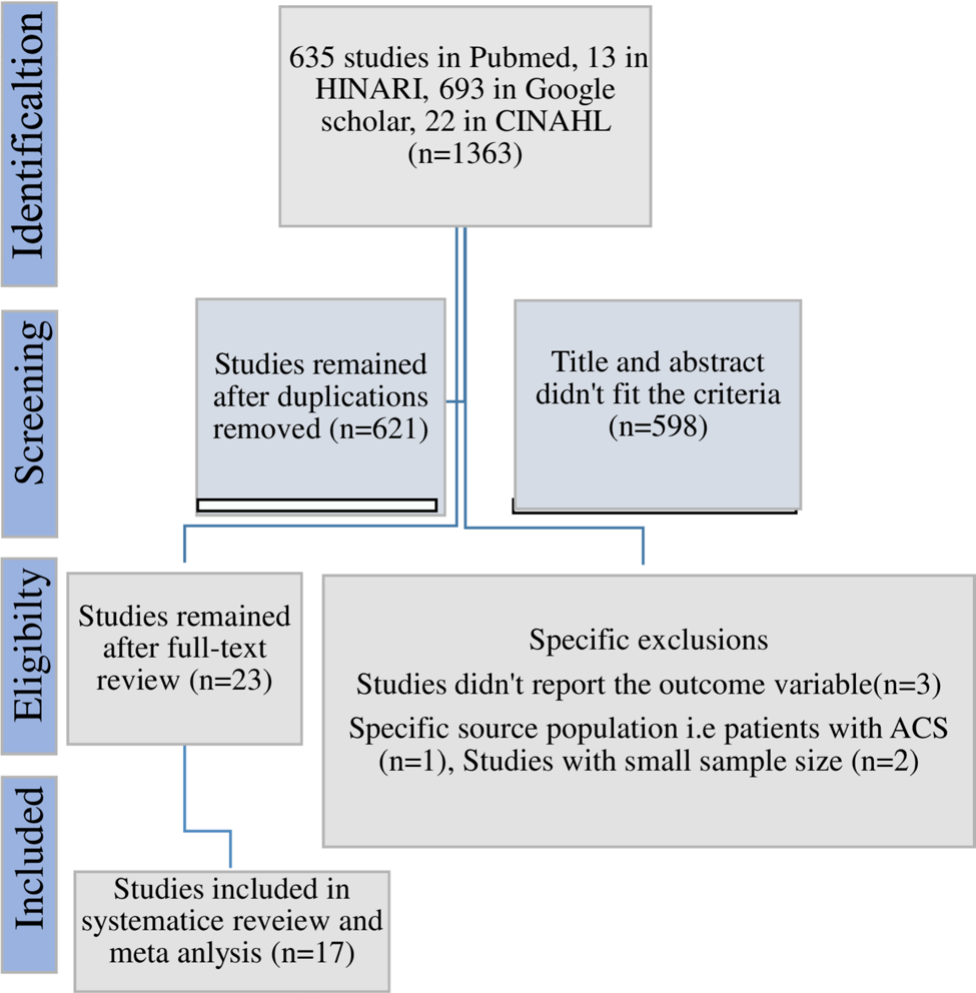
PRISMA flow-chart depicting the selection process of studies in Ethiopia

Using the PRISMA flow diagram, a total of 1363 potentially eligible studies were identified. After removing duplications and conducting a full-text review, 23 studies were retrieved. Finally, 17 studies were included for the final review and Meta-analysis [8, 17–32] (Fig. 1).

### Study characteristics

Studies included in this review and meta-analysis were conducted in Ethiopia and published in indexed journals and/or found as grey literature. Fifteen studies were cross-sectional [8, 18–27, 29–32], and the remaining two were case-control [17, 28]. These studies are conducted in Addis Ababa [8, 18, 19, 21–25, 32], Amhara [20, 27, 30], Oromia [17, 26, 29], South Nation and Nationalities (SNN) [28], and Dire Dawa [31]. The sample size ranged from 175 to 9956. A quality assessment was performed using JBI appraisal tool and studies scored *≥* 50% has considered as low risk of bias. Subsequently, all studies included in this review and meta-analysis have a low risk of bias (Table 1).

**Table 1:** General characteristics of the included studies

Author	Publication year	Region	Age group	Sample size	Prevalence	Study design	Type of population
Tsegaye et al. [28]	2023	SNNP	Pedi	333	11.4	Case control	All
Abebe T et al. [18]	2022	AA	Adult	362	3.6	Cross sectional	All
Hanna D et al. [8]	2021	AA	Adult	506	2.8	Cross sectional	All
Tarkie et al. [27]	2023	Amhara	Adult	5232	1.4	Cross sectional	All
Hunchak et al. [24]	2015	AA	Adult	9956	1.9	Cross sectional	All
Woyessa A et al. [29]	2019	Oromia	Adult	889	8.5	Cross sectional	All
Abebe F et al. [17]	2023	Oromia	Adult	261	28.2	Case control	All
Dode et al. [21]	2022	AA	Adult	388	3.02	Cross sectional	All
Jofira et al. [25]	2018	AA	Pedi	338	4.1	Cross sectional	All
Habte T et al. [19]	2023	AA	Pedi	303	5	Cross sectional	All
Agerie et al. [30]	2022	Amhara	Adult	567	4.6	Cross sectional	All
Fitsumbirhan et al. [23]	2015	AA	Adult	711	3.9	Cross sectional	All
Fikadu A et al. [22]	2021	AA	Adult	331	14.2	Cross sectional	Fall injury
Birhan et al. [20]	2023	Amhara	Adult	415	33	Cross sectional	Trauma
Mamo et al. [26]	2023	Oromia	Adult	357	12.6	Cross sectional	RTI
Tesfaye et al. [32]	2020	AA	Adult	324	3.4	Cross sectional	TBI
Nigussie et al. [31]	2022	Dire Dawa	Adult	175	16.7	Cross sectional	Poisoning

AA; Addis Ababa, RTI; Road Traffic Injury, SNNP; Southern Nation Nationalities and People of Ethiopia, TBI; Traumatic Brain Injury

### Quality and risk of bias assessment

Using the JBI critical appraisal checklist for analytical cross sectional studies, the quality and risk bias of each article included in this review were assessed. All included studies have a low risk of bias as they scored greater than or equal to 50% of the assessment criteria were considered (Table 2).

**Table 2:** Quality and risk of bias assessment of individual studies

No	Checklists	Were the samples representative?	Was the sample size adequate?	Was the exposure measured in a valid and reliable way?	Was appropriate statistical analysis used?	Were confounding factors identified?	Were strategies to deal with confounding factors stated?	Were the outcomes measured in a valid and reliable way?	Was the response rate acceptable	Overall appraisal score
1	Tsegaye et al. [28]	1	1	1	1	1	1	1	1	8
2	Abebe T et al. [18]	1	1	1	1	0	0	1	1	6
3	Hanna D et al. [8]	1	1	1	1	1	1	1	0	7
4	Tarkie et al. [27]	1	1	1	1	N/A	N/A	1	N/A	5
5	Hunchak et al. [24]	1	1	1	1	1	1	1	N/A	7
6	Woyessa A et al. [29]	1	1	1	1	N/A	N/A	1	1	6
7	Abebe F et al. [17]	1	1	1	1	1	1	1	1	8
8	Dode et al. [21]	1	1	1	1	1	1	1	1	8
9	Jofira et al. [25]	1	1	1	1	1	1	1	1	**8**
10	Habte T et al. [19]	1	1	1	1	1	1	1	1	**8**
11	Agerie et al. [30]	1	1	1	1	1	1	1	1	**8**
12	Fitsumbirhan et al. [23]	1	1	1	1	N/A	N/A	1	N/A	**5**
13	Fikadu A et al. [22]	1	1	1	1	1	1	1	1	**8**
14	Birhan et al. [20]	1	1	1	1	1	1	1	1	**8**
15	Mamo et al. [26]	1	1	1	1	1	1	1	1	**8**
16	Tesfaye et al. [32]	1	1	1	1	1	1	1	1	**8**
17	Nigussie et al. [31]	1	1	1	1	N/A	N/A	1	1	**6**

*Note* 1 = Yes 0 = No U = Unclear N/A = Not applicable

### Prevalence of mortality in the ED

**Fig. 2:**
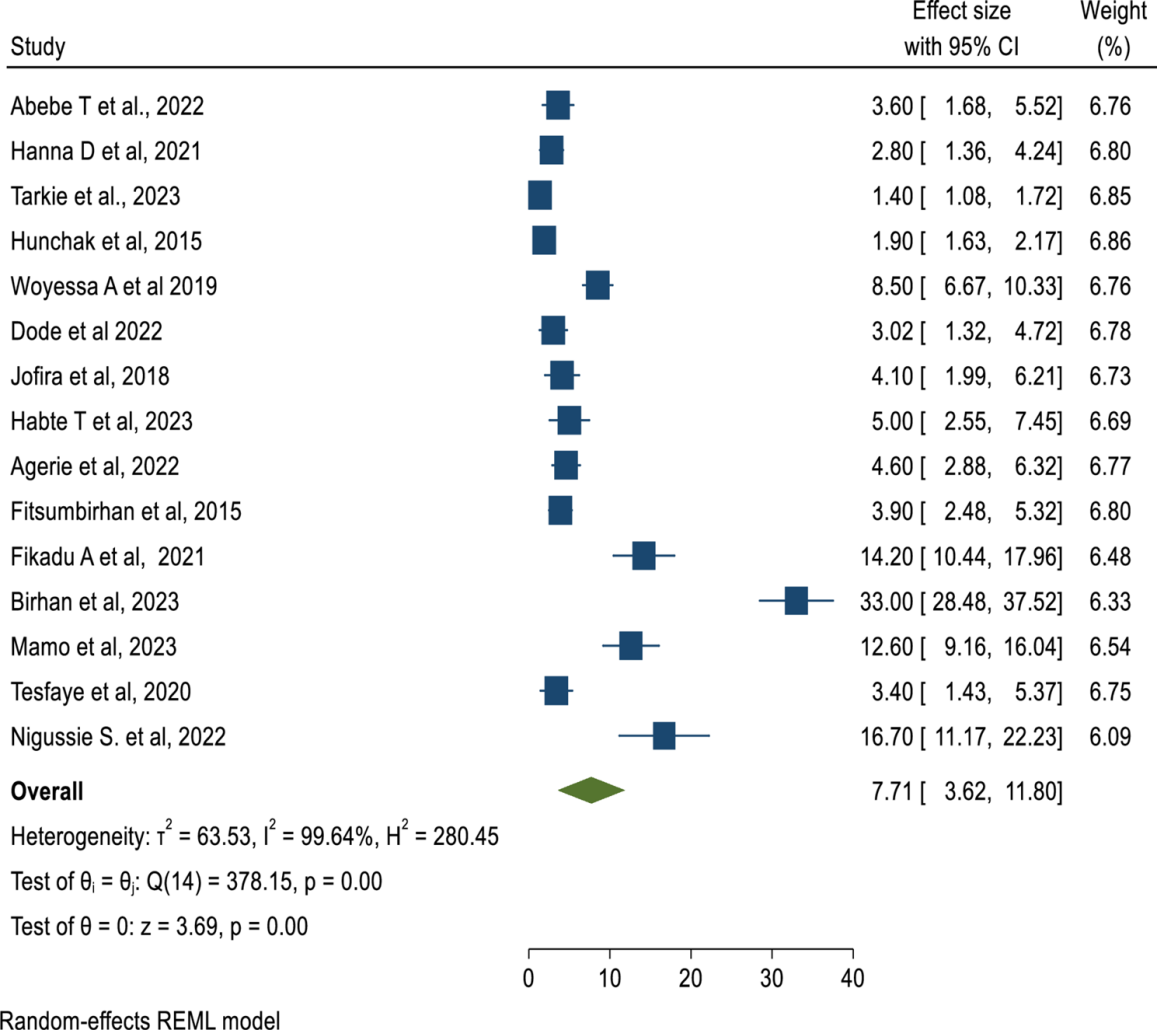
Forest plot to depict the pooled prevalence of mortality in Emergency departments in Ethiopia

In this systematic review and meta-analysis study using a forest plot, the pooled prevalence of mortality of patients in the ED in Ethiopia was found to be 7.71% (95%CI: 3.62, 11.80) which is estimated by a random-effect model (Fig. 2).

### Subgroup analysis

**Fig. 3:**
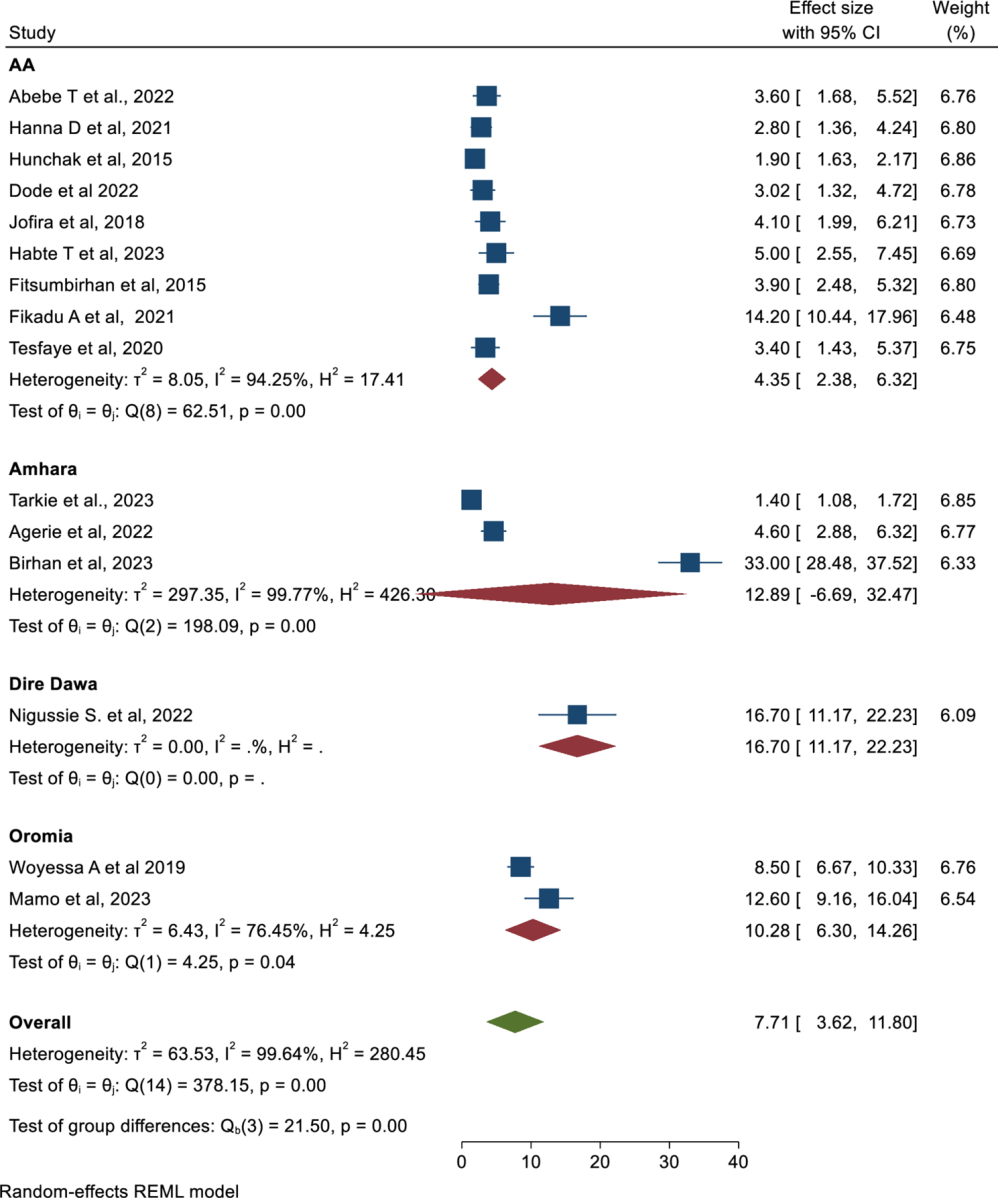
Forest plot to depict the pooled prevalence of mortality in Emergency department by regions in Ethiopia

The subgroup analysis using regions of the country showed that the highest mortality of patients in the ED was observed in Dire Dawa (16.70; 95% CI: 11.17, 22.23), followed by the Amhara region (12.89%; 95% CI: 6.69, 32.47) (Fig. 3).

**Fig. 4:**
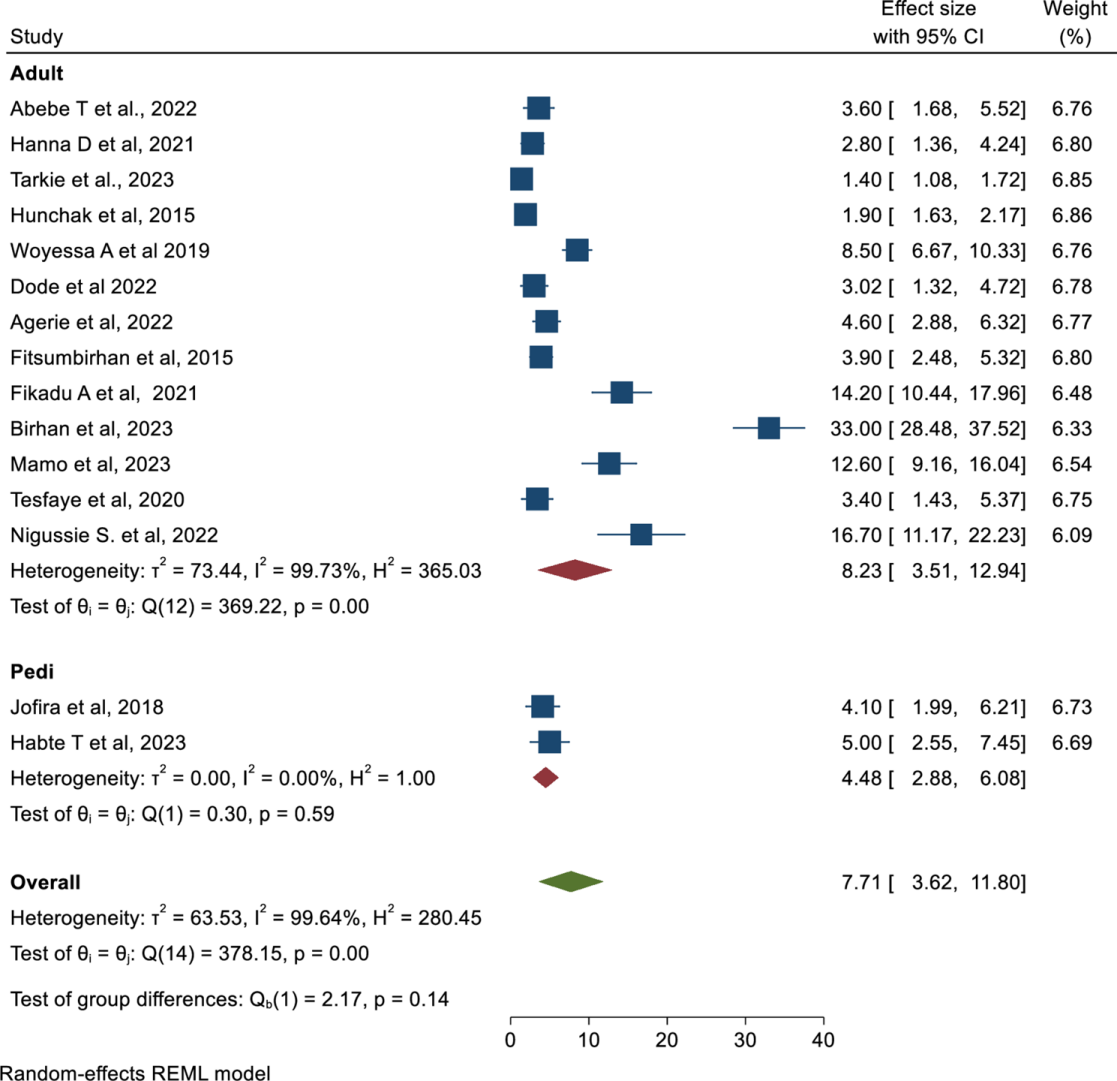
Forest plot to illustrate the pooled prevalence of mortality in Emergency departments by age group in Ethiopia

Similarly, another subgroup analysis using the age of the study population revealed that mortality of patients in the ED among the adults population is higher (8.23; 95% CI: 3.51, 12.94) as compared to pediatrics (4.48%; 95% CI: 2.88, 6.08) (Fig. 4).

**Fig. 5:**
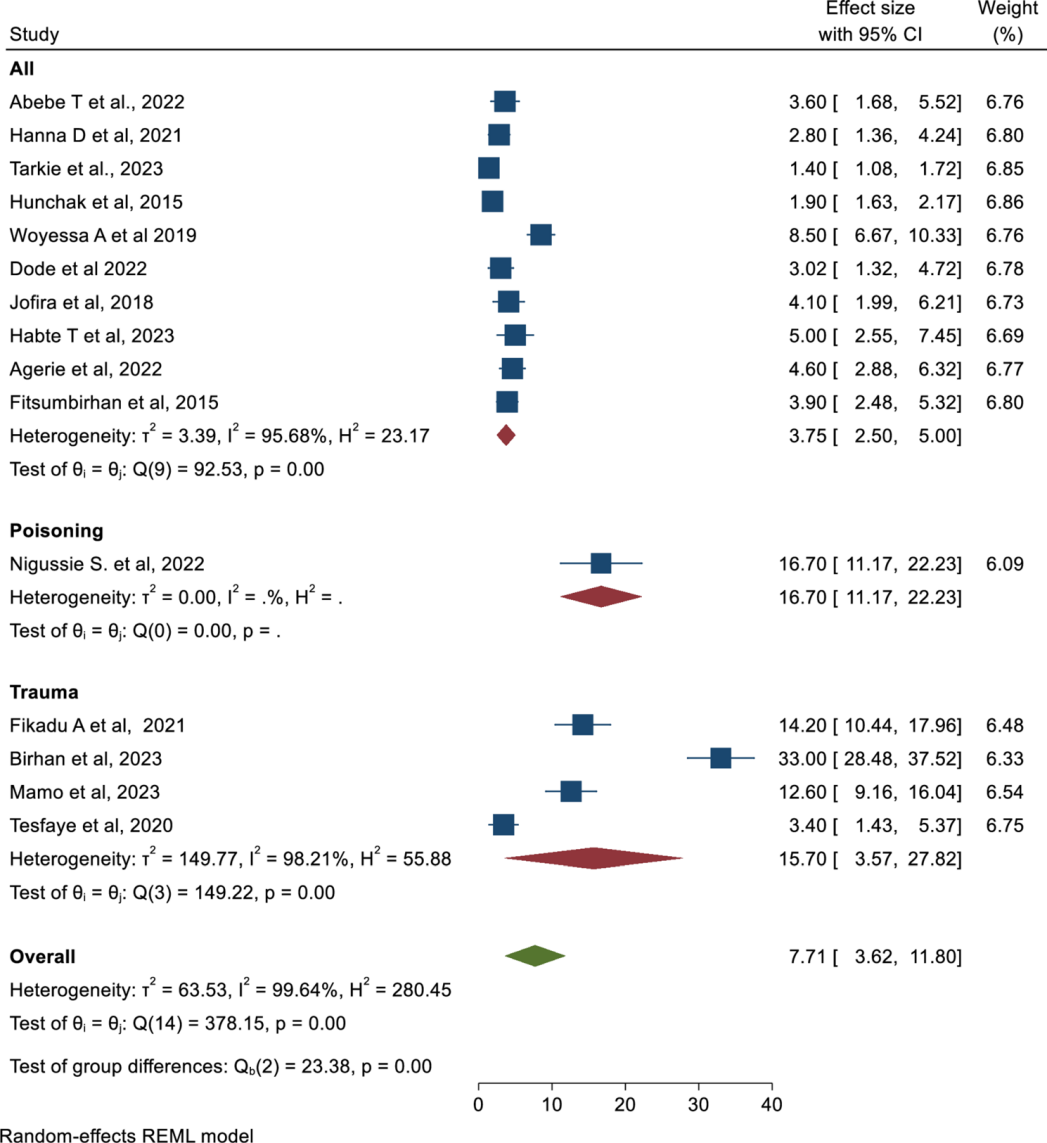
Forest plot to illustrate the pooled prevalence of mortality in Emergency department by type of population in Ethiopia

Furthermore, the subgroup analysis using the type of population studied showed that the highest mortality of patients in the ED was encountered among trauma patients (15.70; 95%CI: 3.57, 27.82) (Fig. 5).

### Publication bias evaluation

**Fig. 6:**
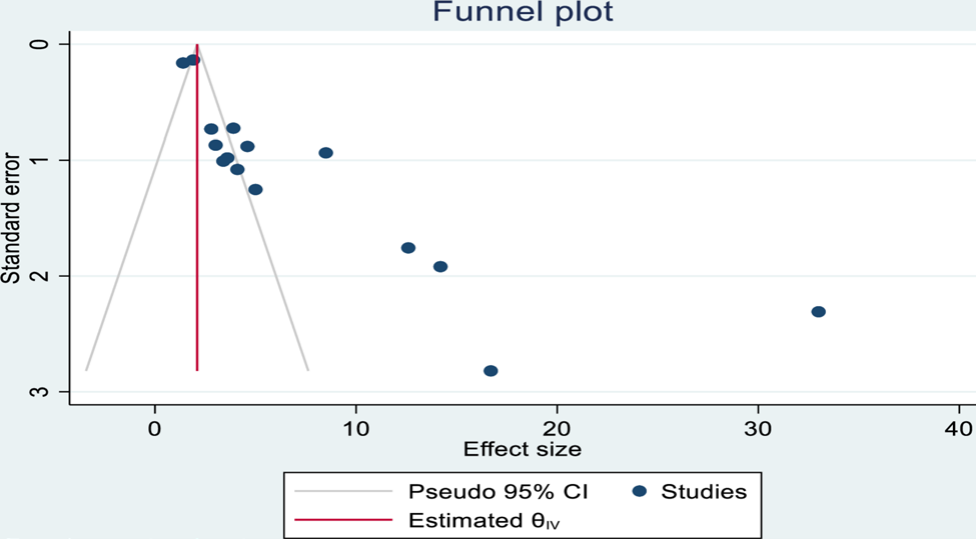
Funnel plot to illustrate the presence of publication bias

The presence of publication bias was evaluated using Egger’s (*p* = 0.000) and Begg’s tests (*p* = 0.003) which showed that there is a significant publication bias. In addition to the statistical tests, a funnel plot was used, and it is asymmetrical. The vertical line indicates the effect size, whereas the diagonal lines indicate the precision of individual studies with a 95% CI (Fig. 6).

### Investigation of heterogeneity

A meta-regression analysis using the sample size and publication year was conducted to identify the possible source of heterogeneity. However, none of these factors showed significant heterogeneity. Therefore, the source of heterogeneity remains unknown (Table 3).

**Table 3:** Meta-regression of mortality of patients in the ED with publication year and sample size to detect heterogeneity

Source of heterogeneity	Coefficients	Standard error	*p*-value
Publication year	1.230673	0.8908009	0.167
Sample size	− 0.0004325	0.0009301	0.642

### Factors associated with mortality of patients in the ED

#### Residence

**Fig. 7:**
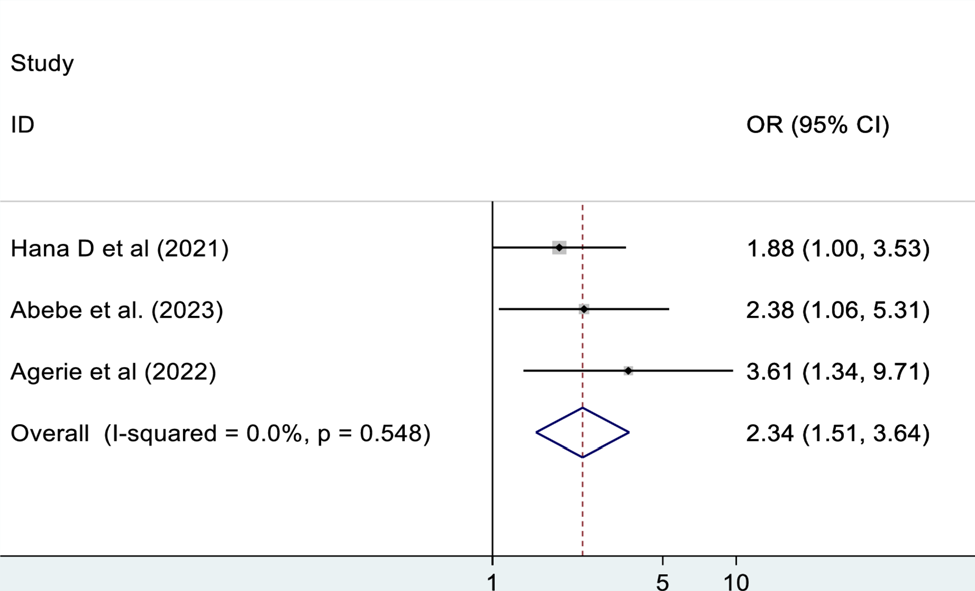
The association between residence and mortality of patients in the ED

The pooled effect of three studies were reported on the association between residence and mortality of patients in the ED. This meta-analysis study revealed that being a rural resident has 2.3 times higher odds of mortality as compared to being an urban resident (OR: 2.30, 95% CI: 1.48, 3.58). There is no observed heterogeneity, I^2^ = 0% (Fig. 7).

#### Unconsciousness

**Fig. 8:**
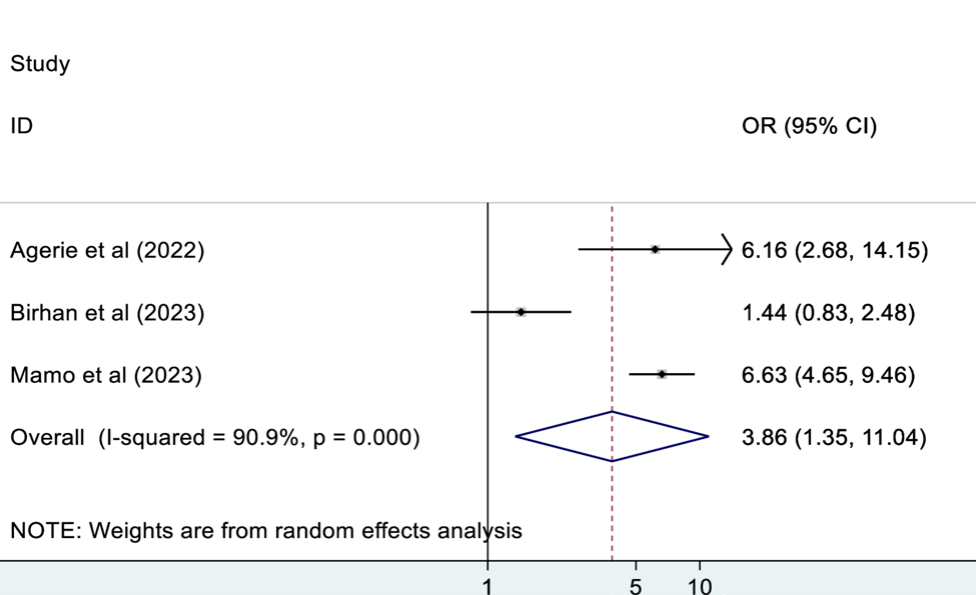
The association between unconsciousness and mortality of patients in the ED

According to this meta-analysis, those patients who were unconscious during admission were 3.9 times more likely to die as compared to their counterparts (OR: 3.86, 95% CI: 1.35, 11.04) (Fig. 8).

#### Comorbidity

**Fig. 9:**
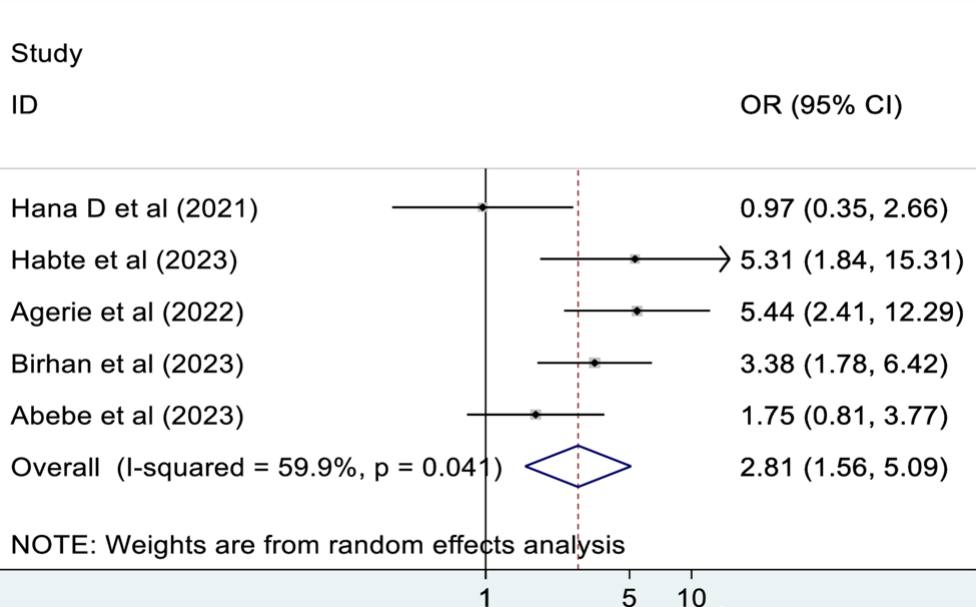
The association between comorbidity and mortality of patients in the ED

The pooled effect of five studies showed that those patients who had comorbidity were 2.8 times more likely to die as compared to those who hadn’t comorbidity (OR: 2.82, 95% CI: 1.56, 5.09). There is a moderate heterogeneity between studies, I^2^ = 59.9% (Fig. 9).

### Time to reach at health facilities

**Fig. 10:**
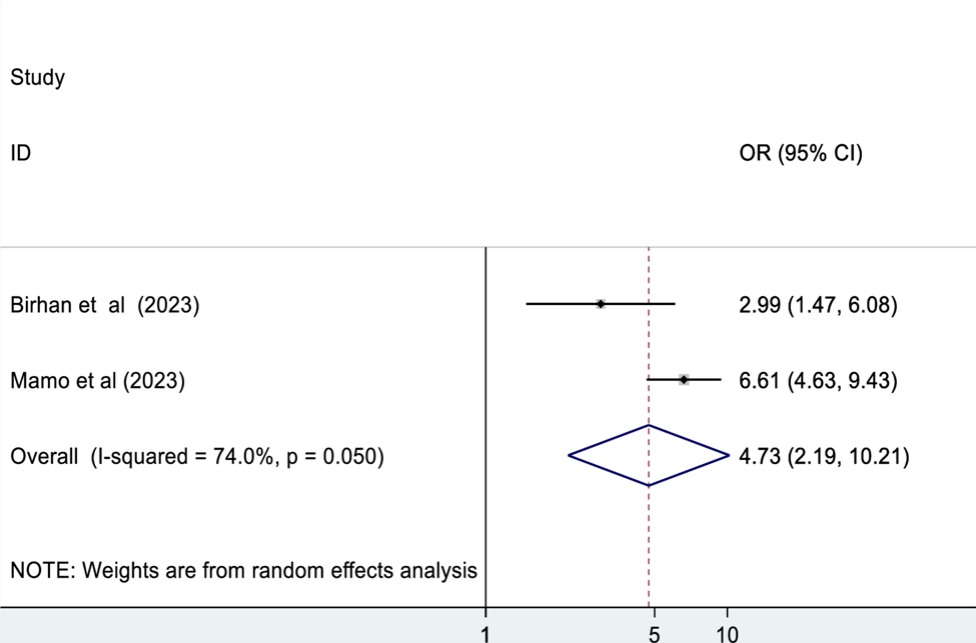
The association between times elapsed to reach health facilities and mortality of patients in the ED

This meta-analysis revealed that the time to reach at the nearby health facilities was another determinant factor associated with mortality of patients in the ED. Those patients who weren’t reached at nearby health facilities within one hour are 4.7 times more likely to die as compared to their counterparts (OR: 4.73, 95% CI: 2.19, 10.21) (Fig. 10 ).

### Sensitivity analysis

A sensitivity analysis using the random effect model showed that the single study didn’t affect the pooled prevalence of mortality (Table 4).

**Table 4:** The sensitivity analysis to estimate the effect of a single study on the combined prevalence of mortality of patients in the ED in Ethiopia

Study omitted	Prevalence (95% CI)
Tsegaye et al. [28]	6.06 (4.85, 7.27)
Hana D. et al. [8]	6.65 (5.37, 7.93)
Hunchak et al. [24]	7.38 (5.52, 9.18)
Woyessa A. et al. [29]	6.12 (4.92, 7.32)
Dode et al. [21]	6.62 (5.34, 7.89)
Jofira et al. [25]	6.52 (5.26, 7.78)
Habte T. et al. [19]	6.45 (5.19, 7.70)
Agerie et al. [30]	6.49 (5.23, 7.75)
Fitsumbirhan et al. [23]	6.56 (5.29, 7.84)
Fikadu et al. [22]	5.93 (4.74, 7.12)
Birhan et al. [20]	5.94 (4.75, 7.12)
Mamo et al. [26]	5.99 (4.79, 7.19)
Tesfaye et al. [32]	6.57 (5.31, 7.84)
Nigussie S. et al. [31]	6.01 (4.1, 7.20)
**Combined**	**7.71 (3.62**,** 11.8)**

## Discussion

Emergency departments’ mortality is one of the major health indices in Ethiopia that should be evaluated monthly in every health institution. Therefore, this systematic review and meta-analysis study is important to generate comprehensive evidence about the prevalence of mortality among patients in EDs and its associated factors in Ethiopia. According to the findings of our study, the pooled prevalence of mortality in the ED was 7.71%. This finding is in line with the studies conducted in England [33] and Nigeria [34], which reported that the overall crud death rate in ED was 8.7% and 7.61%, respectively. However, it is higher as compared to the study conducted in Uganda (2%) [35], Switzerland (0.26%) [36], Sweden (0.88%) [37], England (2.2%) [38], Bulgaria (2.4%) [2], and Denmark (4%) [39]. The possible reasons for this discrepancy might be in industrialized countries, where paramedics begin life-saving measures at the patient’s residence and transport them to the hospital in well-equipped ambulances. In contrast, most patients in our country are brought to the hospital in private cars and traditional ambulances, often without any life-saving interventions before arrival. As a result, many patients arrive with complications and have a little chance of survival [40]. Moreover, it could be due to the differences in the study population, study period, and settings.

According to the current review, the pooled prevalence of mortality among pediatric age groups was 4.48%, which is lower than the study conducted in Burkina Faso [41] (12.9%). The discrepancies might be due to the differences in the study population in terms of disease severity. In addition, it is also higher as compared to the study done in Mexico [42] (21.8%).

This systematic review and meta-analysis revealed that being a rural resident has 2.1 times higher odds of mortality as compared to being an urban resident. This result is supported by the study done in America, which revealed that residents of rural areas experience higher rates of emergency mortality as compared to residents of urban areas [43]. This might be due to the fact that people residing in rural areas have poor health care-seeking behavior and have been presented in the ED lately [44]. Moreover, long distances from health facilities may delay the time to get emergency care, which might potentially lead to high mortality [45].

This meta-analysis study revealed that those patients who were unconscious during admission were 3.9 times more likely to die as compared to their counterparts. This finding is in agreement with studies conducted in Korea [46] and Sweden [47, 48]. The possible reason could be that those unconscious patients are unable to protect their airway, have a high risk of aspiration, have compromised ventilator effort, and are at risk of developing intracranial hypertension, which reduces cerebral perfusion and leads to secondary cerebral attacks and death [49]. Maintaining oxygenation and preventing hypercarbia by preventing aspiration, providing supplemental oxygen, and supporting ventilation are critical in the ED, especially for those unconscious patients [50].

This meta-analysis study revealed that those patients who had comorbidity were 2.8 times more likely to die as compared to those who didn’t have comorbidity. This finding is in harmony with the studies conducted in Iran [3], Sweden [47], and Denmark [39]. This could be due to comorbidities, which have been shown to have a significant impact on clinical course, complications, and patient outcomes [51]. It is a scientific fact that comorbidities such as HIV/AIDS, Diabetes Mellitus (DM), and other comorbidities are immune-compromising diseases. An individual with an immune-compromised condition cannot handle any additional disease burden. In addition, as there is a comorbid disease, patients are more likely to have a greater risk of mortality due to the increased risk of drug side-effects, drug-drug interactions, and drug-disease interactions [52].

According to this meta-analysis, those patients who weren’t reached at nearby health facilities within one hour were 4.7 times more likely to die as compared to their counterparts. This finding is supported by the study conducted in England, which reported that as the time elapsed to reach the health facilities increased by 2 h, the risk of death increased by 1% [53]. Existing evidence indicates that a longer time to reach nearby health facilities leads to delayed presentation to the ED, which can be a detrimental factor for patient mortality [54].

### Limitations of the study

Our study has limitations. We limited the search to English-language articles, so any relevant articles published in foreign languages were not included. We also acknowledge that the studies included in this meta-analysis are observational studies with possible residual confounding, since residual confounding cannot be eliminated in observational studies.

## Conclusion

This study found that the pooled prevalence of mortality of patients in emergency departments of Ethiopian hospitals was high as compared to the Ethiopian Health Sector Transformation Plan II (HSTP II), putting a high healthcare burden on the country, which requires collaboration between all stakeholders to improve outcome. Being a rural resident, unconsciousness, comorbidity, and time elapsed to reach health facilities were significant predictors of mortality. Improving pre-hospital service, expansion of adequately equipped ambulance for patient transportation to point of care, training of health care providers working in the ED, early referral, and improving first line management at referral hospitals will help to reduce the high mortality in our country.

## Data Availability

All data supporting the findings of this study are available within the paper.

## Abbreviations

CI: Confidence Interval
ED: Emergency Department
OR: Odds Ratio
PRISMA: Preferred Reporting Items for Systematic Reviews and Meta-Analyses
WHO: World Health Organization
